# Wild Boars Carry Extended-Spectrum β-Lactamase- and AmpC-Producing *Escherichia coli*

**DOI:** 10.3390/microorganisms9020367

**Published:** 2021-02-12

**Authors:** Anna R. Holtmann, Diana Meemken, Anja Müller, Diana Seinige, Kathrin Büttner, Klaus Failing, Corinna Kehrenberg

**Affiliations:** 1 Institute for Food Quality and Food Safety, University of Veterinary Medicine Hannover, Foundation, Bischofsholer Damm 15, D-30173 Hannover, Germany; anna-ria.holtmann@gmx.de; 2 Working Group Meat Hygiene, Institute Food Safety and Food Hygiene, Freie Universität Berlin, Königsweg 67, D-14163 Berlin, Germany; diana.meemken@fu-berlin.de; 3 Institute for Veterinary Food Science, Justus-Liebig-University, Frankfurter Str. 92, D-35392 Giessen, Germany; anja.mueller@vetmed.uni-giessen.de; 4 Lower Saxony State Office for Consumer Protection and Food Safety, D-26203 Wardenburg, Germany; Diana.Seinige@gmx.de; 5 Unit for Biomathematics and Data Processing, Justus-Liebig-University, Frankfurter Str. 95, D-35392 Giessen, Germany; kathrin.buettner@vetmed.uni-giessen.de (K.B.); klaus.failing@vetmed.uni-giessen.de (K.F.)

**Keywords:** wild boars, ESBL-producing *Escherichia coli*, AmpC, multidrug resistance, MRSA

## Abstract

Extended-spectrum β-lactamase (ESBL)-producing *Escherichia coli* and methicillin-resistant *Staphylococcus aureus* (MRSA) represent major healthcare concerns. The role of wildlife in the epidemiology of these bacteria is unclear. The purpose of this study was to determine their prevalence in wild boars in Germany and to characterize individual isolates. A total of 375 fecal samples and 439 nasal swabs were screened for the presence of ESBL-/AmpC-*E. coli* and MRSA, respectively. The associations of seven demographic and anthropogenic variables with the occurrence of ESBL-/AmpC-*E. coli* were statistically evaluated. Collected isolates were subjected to antimicrobial susceptibility testing, molecular typing methods, and gene detection by PCR and genome sequencing. ESBL-/AmpC-*E. coli* were detected in 22 fecal samples (5.9%) whereas no MRSA were detected. The occurrence of ESBL-/AmpC-*E. coli* in wild boars was significantly and positively associated with human population density. Of the 22 *E. coli*, 19 were confirmed as ESBL-producers and carried genes belonging to *bla*_CTX-M_ group 1 or *bla*_SHV-12_. The remaining three isolates carried the AmpC-β-lactamase gene *bla*_CMY-2_. Several isolates showed additional antimicrobial resistances. All four major phylogenetic groups were represented with group B1 being the most common. This study demonstrates that wild boars can serve as a reservoir for ESBL-/AmpC-producing and multidrug-resistant *E. coli*.

## 1. Introduction

In human and veterinary medicine, the use and misuse of antibiotics has facilitated the emergence of bacteria resistant to antimicrobial agents, including critically important substances like cephalosporins of the third and fourth generation, and fluoroquinolones [1]. Methicillin-resistant *Staphylococcus aureus* (MRSA) and extended-spectrum β-lactamase (ESBL)-producing Enterobacteriaceae are capable of causing severe infections with limited therapy options and are considered to be serious healthcare concerns [2,3]. In 2015, 8750 deaths (median, age-standardized data) in the European Union and European Economic Area were attributed to *Escherichia coli* resistant to third-generation cephalosporins, an approximately 4-fold increase compared to data from 2007 [4]. Farm animals can function as carriers for resistant bacteria, including ESBL-producing *E. coli* and MRSA. Transmission to humans can occur through direct contact or via food products of animal origin [5,6]. Numerous studies in Europe have shown a widespread occurrence of livestock-associated MRSA in farm animals, including cattle, swine, and poultry, as well as in humans that have been exposed through occupations [5,7,8]. Similarly, ESBL-producing *E. coli* has been detected in various domestic animal species [9,10,11]. Additionally, MRSA and ESBL-producing *E. coli* have been detected in liquid manure and dust collected from the surrounding environment of livestock farms [12,13].

Less is known about the role of wildlife in the epidemiology of ESBL-producing *E. coli* and MRSA. Although wild animals are generally not exposed to antimicrobials, they can come into contact with antimicrobial resistant bacteria or antimicrobial residues by foraging and drinking in environments that have been contaminated from anthropogenic sources [14,15]. Several studies have documented that wild animal populations living in close proximity to humans and agricultural areas have a higher prevalence of antimicrobial resistant commensal bacteria than those living in more natural areas [16,17]. Wild boars in particular have been suggested as a potential sentinel species for antimicrobial resistance in wildlife due to their large home ranges, their omnivorous diet, and their high tolerance for human proximity, which can expose them to resistant bacteria or residues of antimicrobial substances [17].

Some studies have detected MRSA or ESBL-producing *E. coli* in game meat [18,19,20] or the feces and tissues of wild animals such as deer, mice, various bird species [21,22], and wild boars [23,24]. This is of particular concern as the handling and consumption of contaminated game meat could constitute a transmission route for resistant bacteria from wild animals to humans. Considering the rising popularity of game meat in Germany and other countries, information about possible reservoirs in free-living wild animal populations are increasingly important in order to identify potential risks for the consumer [25,26]. To date, few studies have selectively investigated the occurrence of MRSA and ESBL-producing *E. coli* in wild boars and examined the molecular characteristics of individual isolates [24,27]. Such information is necessary to gain an understanding of the genotypes present in the wild boar population and to identify possible routes of transmission. Therefore, the purpose of this study was to determine the prevalence of MRSA and ESBL/AmpC-producing *E. coli* in wild boars in Germany, investigate potential risk factors associated with a colonization of wild boars, and to characterize the genetic diversity and antimicrobial susceptibility of the isolates.

## 2. Materials and Methods

### 2.1. Sample Collection

**Figure 1:**
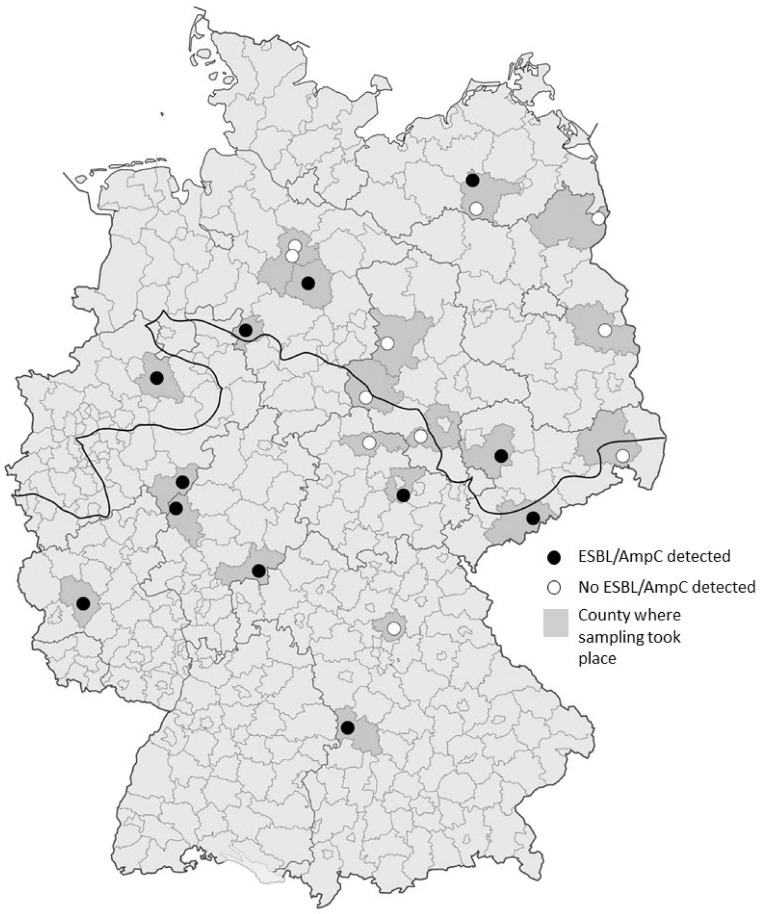
Geographic distribution of sampled hunting locations within Germany. Counties, where sampling took place are shaded in darker grey and the individual hunting locations are represented by circles. Black circles represent locations where ESBL/AmpC-producing *E. coli* were detected, white circles represent locations where all samples tested negative. The fat black line indicates the division between the North German Lowlands and the region of Middle and Southern Germany, including higher altitudes.

Four hundred and thirty-nine (439) nasal swabs and 375 fecal samples were collected from 441 hunted wild boars. The samples were collected during 22 different hunting events from October 2014 to January 2015. The hunting events were organized by professional hunters, foresters, and private hunters and took place in 21 counties across 10 out of 16 federal states in Germany (Figure 1 and Table S1). Age, sex, weight, visual observations of clinical disease (e.g., abscesses, injuries, visible organ lesions), and hunting area were documented for each wild boar (Table S1). The age of wild boars was estimated by assessing tooth eruption and tooth replacement [28]. Individual boars were then assigned to the following age groups: Less than one year old, between one and two years old, older than two years. Nutritional status was recorded based on an evaluation of visual and palpatory body condition [29]. Scores of ≤2 were considered low.

Samples were collected prior to gutting the wild boars. Nasal swabs were collected by inserting sterile swabs deeply into the nares and vigorously rubbing them against the mucosa in rotating motions. Fecal samples were taken directly from the rectum, using sterile spatulas and placed into consecutively numbered sterile tubes. The nasal swabs were placed into Amies Transport Medium (Oxoid Germany GmbH, Wesel, Germany). All samples were immediately cooled to temperatures between 6–8 °C. Subsequently, all samples were transported to the laboratory within 36 h after sampling.

### 2.2. MRSA Isolation

MRSA were isolated based on previously published methods [30,31]. In brief, within 48 h after sampling, nasal swabs were spread on colistin- and nalidixic acid agar (CNA) plates containing 5% (*v*/*v*) sheep blood (Becton Dickinson GmbH, Heidelberg, Germany), selective for streptococci and staphylococci. Subsequently, the nasal swabs were placed in Brain Heart Infusion (BHI)-broth with 6.5% of NaCl. Agar plates and enrichment broth were incubated for 24 h at 36 °C. As considerable growth of contaminating flora was observed on CNA plates in this study, they were excluded from further analyses. To screen the *Staphylococcus aureus* isolates for resistance to β-lactam antibiotics, the inoculated BHI solution was spread on CHROMagar MRSA II agar plates (Becton Dickinson GmbH). Afterwards, the plates were incubated at 36 °C for 24 h and subsequently checked for purple colonies, indicating the presence of presumptive MRSA.

### 2.3. Isolation and Confirmation of ESBL/AmpC-Producing Escherichia coli

Isolation of ESBL/AmpC-producing *E. coli* was performed by selective enrichment, followed by selective plating on chromogenic agar, based on recommendations published by the European Food and Safety Authority [32]. In addition, a non-selective enrichment prior to selective plating was used.

To obtain a 1:10 (*w*/*v*) dilution, one g of each fecal sample was weighed and transferred each into 9 mL of peptone-solution and 9 mL of Mueller-Hinton broth containing 1 mg/L cefotaxime. The suspensions were incubated at 36 °C for 24 h. Subsequently, 1 mL of each suspension was spread on Brilliance ESBL agar plates (Oxoid Germany GmbH, Wesel, Germany). If growth was detectable the following day, a single colony per sample was spread in parallel on Gassner (Oxoid) and on Columbia blood agar (Becton Dickinson GmbH, Heidelberg, Germany) plates, in order to further identify *E. coli* based on expected colony morphology. Isolates that showed blue growth on Gassner agar, indicating lactose-fermentation, and those that produced grey colonies of expected morphology for *E. coli* on Columbia blood agar, were subjected to a final species confirmation using an API 20E test kit (bioMérieux, Nürtingen, Germany). All isolates were stored in cryobank tubes (Mast Diagnosica GmbH, Reinfeld, Germany) at −80 °C.

Confirmation of presumptive ESBL-/AmpC-producing *E. coli* isolates was performed by phenotypic confirmatory testing using ceftazidime (30 µg) and cefotaxime (30 µg) disks (Mast Diagnostica GmbH) with and without the β-lactamase inhibitor clavulanic acid (10 µg). Testing was done by the disk diffusion method according to the Clinical and Laboratory Standards Institute (CLSI) [33]. Isolates were regarded as confirmed ESBL producers if the inhibition zones increased ≥5 mm when the antimicrobials were tested in combination with clavulanic acid and compared to the zone diameters of the cephalosporins alone. Isolates with inhibition zones indicating cefotaxime or ceftazidime resistance during confirmatory testing but no increase in zone diameters in the presence of clavulanic acid were regarded as presumptive AmpC-producers. For quality control, *E. coli* ATCC 25922 and ESBL-producing *Klebsiella pneumoniae* ATCC 700603 were used.

### 2.4. Antimicrobial Susceptibility Testing

The antimicrobial susceptibility of the *E. coli* isolates was determined using the broth microdilution method according to CLSI document M07-A9 [34]. For this, a commercially available microtitre plate (SensititreTM EUVSEC; TREK Diagnostic Systems Ltd., East Grinstead, UK) was used. The following 14 antimicrobial agents were included in the test panel: Ampicillin (concentration range 1–64 µg/mL), azithromycin (2–64 µg/mL), cefotaxime (0.25–4 µg/mL), chloramphenicol (8–128 µg/mL), ciprofloxacin (0.015–8 µg/mL), colistin (1–16 µg/mL), gentamicin (0.5–32 µg/mL), meropenem (0.03–16 µg/mL), nalidixic acid (4–128 µg/mL), sulfamethoxazole (8–1024 µg/mL), ceftazidime (0.5–8 µg/mL), tetracycline (2–64 µg/mL), tigecycline (0.25–8 µg/mL), and trimethoprim (0.25–32 µg/mL). *Escherichia coli* ATCC 25922 was used as a quality control strain. The microtiter plates were incubated at 36 °C for 18 h. Minimal inhibitory concentrations (MIC) were interpreted according to CLSI document M100S [33]. As there are no CLSI-approved colistin breakpoints available for *E. coli*, those provided by the European Committee on Antimicrobial Susceptibility testing (EUCAST) were used. For azithromycin and tigecycline, for which there are no accepted breakpoints available for *E. coli*, isolates with MIC values of >16 µg/mL azithromycin and >2 µg/mL tigecycline were considered to be resistant.

### 2.5. DNA Isolation, Species Confirmation and PCR Analysis

Genomic DNA was extracted by using the DNeasy blood and tissue kit (Qiagen, Hilden, Germany). PCR analyses were carried out using 25 µL reaction mixtures containing 2 µL template DNA, 0.4 µM each of forward and reverse primers, 2.5 mM MgCl_2_, 1 U *Taq*-DNA-Polymerase (Invitrogen; Thermo Fisher Scientific, Dreieich, Germany), and 2.5 µL corresponding 10xPCR buffer. Cycling conditions were used based on the protocols published for individual primer pairs. Confirmed ESBL-producing *E. coli* were tested for the presence of the β-lactamase encoding genes *bla*_TEM_, *bla*_SHV_, and *bla*_CTX-M_ using previously described PCR assays [35]. Isolates yielding positive results with the CTX-M universal primers were further tested with a set of group-specific primer pairs targeting CTX-M groups 1, 2, 8, 9, and 25 [36,37]. In order to determine the specific β-lactamase types, amplicons of *bla*_TEM_, *bla*_SHV_ and *bla*_CTX-M_ groups were sequenced using the TubeSeq sequencing service provided by Eurofins Genomics (Ebersberg, Germany). Presumptive AmpC-producing isolates were tested for the AmpC β-lactamase gene *bla*_CMY-2_ [38]. All isolates carrying *bla*_CMY-2_ were regarded as confirmed AmpC producers.

The detection of additional resistance determinants was performed using a set of PCR assays as described previously [39]. This included determinants mediating resistance to tetracyclines (*tet*(A), *tet*(B), *tet*(C), *tet*(D), *tet*(E), *tet*(G), and *tet*(H)), sulfonamides (*sul1*, *sul2* and *sul3*), trimethoprim (*dfrA5/dfrA14*, *dfrA7/dfrA17*, *dfrA1*, and *dfrB1/dfrB2*) phenicols (*cmlA*, *floR*) and those associated with resistance to quinolones (*qnrA*, *qnrB*, *qnrC*, *qnrD*, *qnrS*, *qepA*, *aac(6′)-Ib-cr*, *gyrA*, *gyrB*, *parC, parE*). Amplicons of *dfrA5/dfrA14*, *dfrA7/dfrA17* or *gyrA*, *gyrB*, *parC*, and *parE* were sequenced (Eurofins Genomics). All obtained sequencing data were analyzed using the BLAST-algorithm (http://www.ncbi.nlm.nih.gov/BLAST/, last accessed 3 December 2020) [40].

*E. coli* isolates were assigned to the four major phylogenetic groups A, B1, B2 and D using PCR assays targeting the genes *chuA* and *yjaA* and the DNA fragment TSPE4.C as described previously [41].

### 2.6. Genotyping

Pulsed-field gel electrophoresis (PFGE) was used to further establish the genetic relatedness of the *E. coli* isolates. For digestion of genomic DNA, the restriction enzyme *Xba*l was used. Restriction fragments were separated in a CHEF DR II system (BioRad, Munich, Germany) using a 0.5 tris-borate-EDTA buffer. The pulse time was increased from 2 s to 20 s during the entire run-time of 20 h. Analysis of fragment patterns was done by using the BioNumerics software (version 7.0; Applied Maths, Sint-Martens-Latem, Belgium). The resulting band patterns were processed using the dice coefficient with 0.5% optimization and 1% position tolerance.

### 2.7. Genome Sequencing

Seven *E. coli* isolates with low similarity of PFGE band patterns were selected for whole genome sequencing. These isolates were chosen to include isolates with varying phylogenetic groups, β-lactamase genes and other resistance genes. Genome sequencing was provided by MicrobesNG (http://www.microbesng.uk, last accessed: 18 May 2020), which is supported by the BBSRC (grant number BB/L024209/1). Genome sequencing was used to define multilocus sequence types (ST) of the isolates.

### 2.8. Statistical Analyses

Statistical analyses were performed by means of the statistical program package SAS 9.4 [42] to evaluate associations between the ESBL- and AmpC-status of the samples and the variables sex, age group, weight, and nutritional status of the wild boars as well as the sampling region and population density of the counties (people/km^2^, based on population data for 2018 provided by the statistical offices of the federal states, available at https://www.statistischebibliothek.de [last accessed 10 October 2020], and area of the county). The population density data showed a right-skewed distribution, so they were logarithmically transformed prior to the analyses. Logistic regression analyses were performed in order to determine the effect of each independent variable separately (sex, age group, weight, nutritional status, population density) on the ESBL- and AmpC-status of the samples.

## 3. Results

### 3.1. MRSA and ESBL/AmpC-E. coli Isolate Detection

Presumptive ESBL-producing *E. coli* were detected in 24 of the 375 fecal samples. Of these, 19 isolates were determined as ESBL-producers following confirmatory testing. Another three isolates were identified as AmpC-producers. The overall prevalence of ESBL-/AmpC-producing *E. coli* in wild boars was 5.9% (22/375; 95% CI, 3.5%–8.3%). ESBL-/AmpC-positive samples were found across all age groups, both sexes, and sampling regions (Table 1). A statistically significant, positive association (*p* < 0.05) was observed between ESBL- and AmpC-positive samples and a higher population density of the county (Table 2). No significant associations were observed for sex, weight, age group, nutritional status, or region of sampling (Table 2). In contrast, MRSA was not detected in any of the nasal swabs.

### 3.2. Antimicrobial Susceptibility Testing and Detection of Resistance Genes

**Figure 2:**
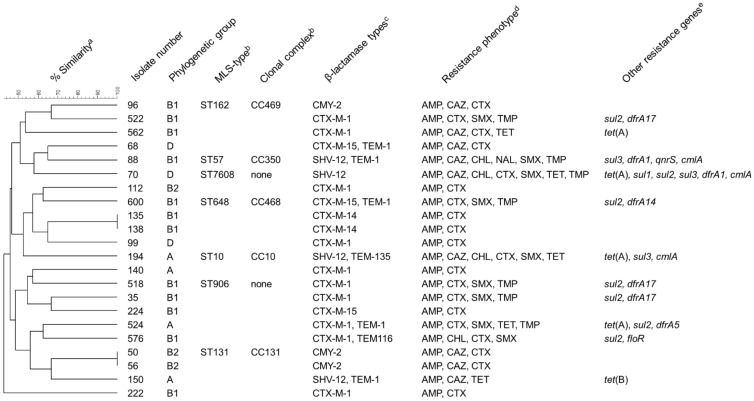
Characteristics of *E. coli* isolates collected in the course of the present study. ^a^ Dendrogramm depicting the relatedness of the isolates and the percentage of similarity based on PFGE band patterns after *Xba*I digestion. ^b^ Multilocus sequence types and clonal complexes according to genome sequences. These data are only available for isolates subjected to genome sequencing. None: ST does not belong to a defined clonal complex. ^c^ Includes ESBLs, AmpC-β-lactamases and additional non-extended-spectrum β-lactamases (TEM-1, TEM-116, TEM-135). ^d^ Resistance phenotype based on MICs determined by broth macrodilution susceptibility testing and evaluation according to CLSI breakpoints for *E. coli*. AMP = ampicillin, CAZ = ceftazidime, CHL = chloramphenicol, CTX = cefotaxime, NAL = nalidixic acid, SMX = sulfamethoxazole, TET = tetracyclines, TMP = trimethoprim. ^e^ Resistance genes other than β-lactamase genes detected in the isolates by PCR.

The phenotypic resistance patterns and according genotypes of all isolates are shown in Figure 2. Phenotypic susceptibility testing showed that of the 22 *E. coli* isolates, all were resistant to ampicillin (MIC ≥ 32 µg/mL), 20 isolates to cefotaxime (MIC ≥ 4 µg/mL), and 9 isolates to ceftazidime (MIC ≥ 16 µg/mL) (Figure 2). Overall, half of the isolates (*n* = 11) were resistant to β-lactams only.

The genes of the CTX-M group (15 isolates) were the most prevalent ESBL genes detected in the isolates using PCR and sequencing, with *bla*_CTX-M-1_ being the most commonly detected gene on these 15 isolates. The remaining four confirmed ESBL-producers carried *bla*_SHV-12_. All three AmpC-producers harbored *bla*_CMY-2_. Seven isolates carried an additional non-extended-spectrum β-lactamase of the TEM family (Figure 2).

In addition to β-lactam resistance, the most common resistances were to sulfonamides (MIC ≥ 512 µg/mL, *n* = 9), trimethoprim (MIC ≥ 16 µg/mL, *n* = 7), tetracyclines (MIC ≥ 16 µg/mL, *n* = 5), chloramphenicol (MIC ≥ 32 µg/mL, *n* = 4), and nalidixic acid (MIC ≥ 32 µg/mL, *n* = 1). Overall, 13 isolates were resistant to at least one additional class of antimicrobial agents besides β-lactams, of which five were multiresistant (resistant to at least three classes of antimicrobials). No isolates were resistant to azithromycin, colistin, gentamicin, meropenem, or tigecycline.

The most common resistance genes detected were those mediating resistance to sulfonamides, including *sul1* (*n* = 1), *sul2* (*n* = 7), and *sul3* (*n* = 3), and trimethoprim, including *dfrA1* (*n* = 2), *dfrA5* (*n* = 1), *dfrA14* (*n* = 1) or *dfrA17* (*n* = 3) (Figure 2). One isolate carried all three of the sulfonamide genes. Among the tetracycline-resistant isolates, *tet*(A) was the most common resistance determinant (*n* = 4), whereas *tet*(B) was identified in only one isolate (Figure 2). The phenicol resistance genes *cmlA* and *floR* were detected in three and one isolate, respectively (Figure 2). The isolate showing resistance to nalidixic acid and ciprofloxacin carried *qnrS* (Figure 2). In addition, mutations in the quinolone-resistance determining region of *gyrA* were identified in this isolate, leading to a Ser83Leu exchange.

### 3.3. Typing

The assignment to phylogenetic groups of the 22 *E. coli* isolates revealed that 12 out of 22 isolates belonged to the phylogenetic group B1 and 4 isolates belonged to group A. Three isolates belonged to each of groups D and B2 (Figure 2).

In total, 20 different macrorestriction patterns were detected (Figure S1). In two cases, two isolates showed indistinguishable band patterns. These isolates were collected during the same hunt in North-Rhine Westphalia or the federal state Hesse, respectively (Figure 2). No clustering (≥80% similarity) was observed among the remaining 18 isolates [43].

Whole-genome sequencing of the seven isolates revealed the following multilocus sequence types: ST10, ST57, ST131, ST162, ST648, ST906, and a novel sequence type that has been assigned to ST7608 in the EnteroBase Escherichia/Shigella database (http://enterobase.warwick.ac.uk/, last accessed: 4 December 2020).

## 4. Discussion

We demonstrated that wild boars hunted at various sites across Germany carry ESBL/AmpC-producing *E. coli* in their feces. The ESBL/AmpC-carriers comprised animals of all age groups as well as of both sexes. The observed prevalence in our study is much lower than that reported for livestock such as fattening pigs, of which approximately 46% carry ESBL/AmpC producers in Germany [44]. The use of antimicrobial agents in intensive livestock farming is a significant driver of the development and spread of antimicrobial resistance [45]. As wild boars are generally not treated with these substances, a lower selection pressure for the development of antimicrobial resistance can be expected and the presence of resistant bacteria is likely a result of environmental contamination [17]. As one of the most frequently hunted wild animals in Germany, wild boar meat constitutes a significant proportion of game meat. Nearly 500,000 individuals were harvested in the hunting season of 2014/2015, when our sampling took place [46] and a transmission of ESBL/AmpC- producing *E. coli* from wild boars to consumers along the food chain might occur. In addition, the presence of resistant bacteria in wildlife is an indicator of the distribution of antimicrobial resistance from anthropogenic sources to the environment [17]. This is supported by the significant association observed in our study, between ESBL/AmpC-positive samples and higher population densities of the counties. One possibility of how wild animals, especially burrowing wild boars, could be colonized or infected with resistant bacteria is through contact with contaminated ground or surface water while feeding and drinking. Friese et al. [13] reported the detection of MRSA and ESBL-producing bacteria in soil samples from fields fertilized with pig manure and other studies found that these bacteria can be dispersed through the air and land on the ground in the vicinity of pig farms [47,48]. Wild boars in particular display a high tolerance for human proximity [17]. In fact, this tolerance can cause a number of problems. Conflicts between humans and wild boars may arise especially due to the destruction of agricultural crops, public green areas, or private property, which may cause significant economic losses [49]. They do not even shy away from heavily populated areas such as the city of Berlin [50]. In these urban environments, they may feed on anthropogenic food sources and waste, which can potentially expose them to traces of antimicrobial substances or directly to antimicrobial resistant bacteria [17]. They can then function as possible reservoirs for antimicrobial resistant bacteria and may facilitate their further dispersal. In addition, they can carry diseases, which may threaten livestock animals. In particular, the first detection of African swine fever virus in wild boars hunted in Germany near the border to Poland in 2020 has raised serious concerns about a transmission to domestic pigs [51].

Our detection rate of ESBL/AmpC-producing *E. coli* of 5.9% (3.5–8.3%) is within range of what has been reported previously in wild boars in Spain (10%) [24], Poland (2.7%, range 1.0–4.5%) [52] and the Czech Republic (2%) [53]. In 2016, samples from the feces of wild boars were included in the German national antimicrobial resistance monitoring and the reported prevalence of 6.4% (4.6–8.7%) ESBL-/AmpC-producing *E. coli* in fecal samples was very similar to our results [54]. The following year, fecal samples of roe deer were tested, showing a lower prevalence compared to wild boars with 2.3% (1.3–3.9%) positive samples [55]. When comparing prevalence, however, differences in sampling strategies, detection methods, and confirmatory testing have to be considered. Poeta et al. [24] and Wasyl et al. [52] directly streaked fecal samples onto selective agar without prior enrichment, which may have reduced sensitivity of detection. In contrast, Literak et al. [53] collected rectal swabs from wild boars, which were incubated in non-selective pre-enrichment medium, followed by selective enrichment prior to plating, similar to the method used for fecal samples examined in the national monitoring [53,54].

In Europe, *bla*_CTX-M-1_, *bla*_TEM-52_ and *bla*_SHV-12_ are the most frequently detected β-lactamase types in isolates of animal origin [56]. The majority of 3rd generation cephalosporin-resistant isolates from pig farms in Germany carry the ESBL gene *bla*_CTX-M-1_ and other genes are detected less frequently [5,57,58]. Among the isolates in the present study, *bla*CTX-M-1 was also the most commonly detected ESBL gene. The isolates in other studies on wild boars also often carried *bla*_CTX-M-1_ or *bla*_TEM-52b_, and a single isolate in Poland carried *bla*_CTX-M-15_ [24,52,53]. Wasyl et al. also detected *bla*_CMY-2_ in 6 of 9 cephalosporin-resistant *E. coli*, whereas no AmpC-producing isolates were reported in the other two studies. Similar to our results for isolates from wild boars, previous studies found a great variety of sequence types among ESBL/AmpC-producing *E. coli* from domestic pigs in Germany, however, ST10 and ST88 seemed to be among the most frequent STs [5,57,58]. Isolates belonging to ST131, frequently producing *bla*_CTX-M-15_, are most commonly associated with carriage and infections in humans [56,57]. The isolate carrying *bla*_CTX-M-15_ in this study, for which a sequence type was determined, belonged to ST648, however. Sequence type 648/CTX-M-15 isolates have previously been associated with companion animals [59]. The isolate belonging to ST131 in the current study carried the AmpC-β-lactamase gene *bla*_CMY-2_. This isolate, as well as two further isolates, belonged to phylogenetic group B2. This group, and to a lesser extent group D, comprise most extraintestinal pathogenic *E. coli*. Only three isolates belonged to group A, which contains mostly commensal strains [41]. Other studies of ESBL-producing *E. coli* in free living wild boars most frequently observed the phylogenetic group A (5/5) [53] or phylogenetic group B1 (3/8) and B2 (3/8) [24]. Similar to our results, resistances to sulfonamides, trimethoprim, and tetracycline, were also commonly observed in ESBL producers and resistant indicator *E. coli* from wild boars examined in previous studies [24,52,53,60]. The isolates in these studies were also frequently resistant to streptomycin, which was not included in the test panel in the current study, and higher rates of resistances to quinolones were determined compared to our results.

While ESBL/AmpC-producing *E. coli* were detected in sampled wild boars across Germany, no MRSA were isolated from nasal swabs. In one study conducted in Spain, researchers were able to isolate MRSA from three nasal swabs and five skin swabs of 817 wild boars [27] and in another study, a single CC398 MRSA was detected among *S. aureus* recovered from mouth and nose samples from wild boars in Portugal [23]. In other studies, in Germany, MRSA was not detected in nasal swabs of wild boars using cultural methods, either [30,31]. Overall, it appears that MRSA are rarely present in wild boars in Europe.

## 5. Conclusions

The results of this study demonstrate that wild boars in Germany may constitute a reservoir for the dissemination of ESBL- and AmpC-producing *E. coli* and that proximity to areas densely populated by humans appears to result in higher detection rates. Further research, including longitudinal studies over multiple years, is necessary to further explore the association between anthropogenic activity and the occurrence of resistant strains in wild boars as well as the potential of wild boars to transmit these bacteria to humans.

## Figures and Tables

**Table 1:** Observed frequencies of qualitative characteristics and averages of quantitative attributes of Extended-spectrum β-lactamase (ESBL)- and AmpC β-lactamase-positive and -negative wild boars and their respective harvesting locations.

	ESBL/AmpC-Positive	ESBL/AmpC-Negative
**Qualitative data**		
**Sex**		
Male (*n* = 187)	8.0% (4.5–12.9%, *n* = 15)	92.0% (87.1–95.4%, *n* = 172)
Female (*n* = 186)	3.8% (1.5–7.6%, *n* = 7)	96.2% (92.4–98.5%, *n* = 179)
**Age group (years)**		
0–1 (*n* = 197)	6.1% (3.2–10.4%, *n* = 12)	93.9% (89.6–96.8%, *n* = 185)
1–2 (*n* = 99)	5.1% (1.7–11.4%, *n* = 5)	94.9% (88.6–98.3%, *n* = 94)
>2 (*n* = 78)	6.4% (2.1–14.3%, *n* = 5)	93.6% (85.7–97.9%, *n* = 73)
**Nutritional status**		
Normal (*n* = 334)	5.7% (3.5–8.7%, *n* = 19)	94.3% (91.3–96.5%, *n* = 315)
Low (*n* = 41)	7.3% (1.5–19.9%, *n* = 3)	92.7% (80.1–98.5%, *n* = 38)
**Sampling Region**		
Northern German Lowlands (*n* = 181)	5.5% (2.7–9.9%, *n* = 10)	94.5% (90.1–97.3%, *n* = 171)
Middle & Southern Germany (*n* = 194)	6.2% (3.2–10.6%, *n* = 12)	93.8% (89.4–96.7%, *n* = 182)
**Quantitative data**		
Average weight (kg)	40.95 (28.86–53.04)	39.03 (36.68–41.38)
Average population density of county (people/km^2^)	192.64 (158.67–226.6)	149.26 (140.05–158.47)

95% confidence intervals are given in parentheses.

**Table 2:** Statistical associations of different attributes and the detection of ESBL-/AmpC-producing *E. coli* in fecal samples of wild boars.

Effect	Odds Ratio	95% CI	*p*-Value
Sex: male (vs. female)	2.23	0.89–5.60	0.088
Age:			
<1 year (vs. 1–2 years)	1.22	0.42–3.56	0.717
>2 years (vs. <1 year)	1.06	0.36–3.10	0.921
1–2 years (vs. >2 years)	0.78	0.22–2.78	0.698
Nutritional status: low (vs. normal)	1.31	0.37–4.63	0.676
Region: north (vs. mid/south)	1.13	0.48–2.68	0.786
Population density ^1^	8.67	1.45–51.89	0.018
Weight	1.00	0.98–1.02	0.708

^1^ Based on logarithmically transformed data; Reference groups are given in parentheses; CI = confidence interval.

## Data Availability

Not applicable.

## Supplementary Materials

The following are available online at https://www.mdpi.com/2076-2607/9/2/367/s1, Figure S1: Dendrogram and similarity of isolates based on *Xba*I macrorestriction analysis including band patterns of individual isolates. Table S1: Attributes of sampled wild boars.
