# Supplementary figures and images for: Wild Boars Carry Extended-Spectrum β-Lactamase- and AmpC-Producing Escherichia coli
Source: Microorganisms. 2021 Feb 12;9(2):367. doi: 10.3390/microorganisms9020367 (PMC7917586; doi:10.3390/microorganisms9020367)

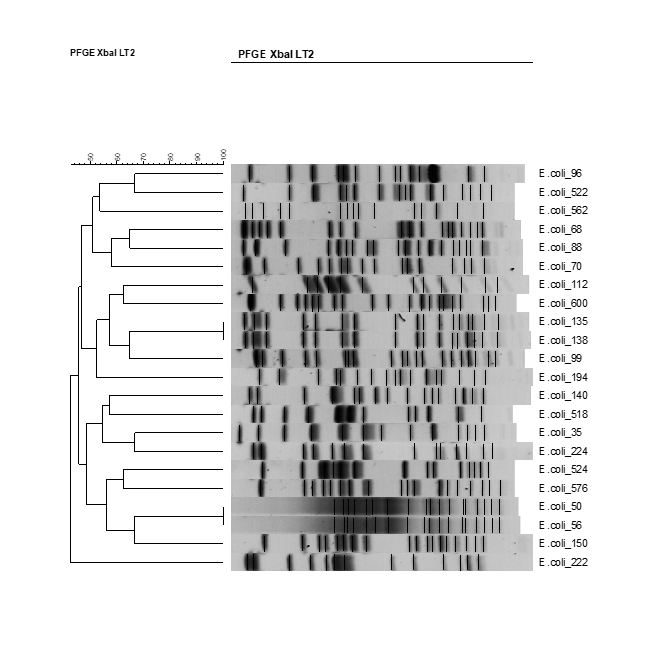

Supplement: Supplementary file 1 [file microorganisms-09-00367-s001.zip › Microorganisms 2021 Figure S1.tif]
